# Metabolic reprogramming of pulmonary fibrosis

**DOI:** 10.3389/fphar.2022.1031890

**Published:** 2022-11-14

**Authors:** Jiaxin Li, Xiaoxuan Zhai, Xiao Sun, Shengchuan Cao, Qiuhuan Yuan, Jiali Wang

**Affiliations:** ^1^ Department of Emergency Medicine, Qilu Hospital of Shandong University, Jinan, China; ^2^ Shandong Provincial Clinical Research Center for Emergency and Critical Care Medicine, Institute of Emergency and Critical Care Medicine of Shandong University, Chest Pain Center, Qilu Hospital of Shandong University, Jinan, China; ^3^ Key Laboratory of Emergency and Critical Care Medicine of Shandong Province, Key Laboratory of Cardiopulmonary-Cerebral Resuscitation Research of Shandong Province, Shandong Provincial Engineering Laboratory for Emergency and Critical Care Medicine, Qilu Hospital of Shandong University, Jinan, China; ^4^ The Key Laboratory of Cardiovascular Remodeling and Function Research, Chinese Ministry of Education, Chinese Ministry of Health and Chinese Academy of Medical Sciences, The State and Shandong Province Joint Key Laboratory of Translational Cardiovascular Medicine, Qilu Hospital of Shandong University, Jinan, China

**Keywords:** pulmonary fibrosis, metabolic reprogramming, glucose metabolism, lipid metabolism, amino acid metabolism, myofibroblasts

## Abstract

Pulmonary fibrosis is a progressive and intractable lung disease with fibrotic features that affects alveoli elasticity, which leading to higher rates of hospitalization and mortality worldwide. Pulmonary fibrosis is initiated by repetitive localized micro-damages of the alveolar epithelium, which subsequently triggers aberrant epithelial-fibroblast communication and myofibroblasts production in the extracellular matrix, resulting in massive extracellular matrix accumulation and interstitial remodeling. The major cell types responsible for pulmonary fibrosis are myofibroblasts, alveolar epithelial cells, macrophages, and endothelial cells. Recent studies have demonstrated that metabolic reprogramming or dysregulation of these cells exerts their profibrotic role *via* affecting pathological mechanisms such as autophagy, apoptosis, aging, and inflammatory responses, which ultimately contributes to the development of pulmonary fibrosis. This review summarizes recent findings on metabolic reprogramming that occur in the aforementioned cells during pulmonary fibrosis, especially those associated with glucose, lipid, and amino acid metabolism, with the aim of identifying novel treatment targets for pulmonary fibrosis.

## Introduction

Pulmonary fibrosis (PF), a progressive and intractable lung disease that affects alveoli elasticity, is characterized by exaggerated inflammatory and fibrotic responses that lead to pulmonary fibrotic remodeling and extracellular matrix deposition, which in turn perpetuates fibrosis formation in a vicious cycle of fibrosis formation (Distler et al., 2019). Idiopathic pulmonary fibrosis (IPF), the classic and most common type, which accounts for 20% of Interstitial lung diseases, is a chronic progressive and irreversible lung disease that usually rapidly progresses to respiratory failure and death (median interval from diagnosis to death is 3 years (Lederer and Martinez, 2018). It has been estimated that the overall prevalence of PF in European countries is about 75 cases per 100,000 people (Wijsenbeek and Cottin, 2020). The high incidence and serious clinical outcomes of PF have prompted a deep exploration of PF’s pathogenesis.

It has been well established that damage and/or dysfunction of alveolar epithelial (AE) cells occurs during the initial stage of the disease (Kasper and Haroske, 1996; Winters et al., 2019; Moss et al., 2022). Then the dysregulated AE cells interact with mesenchymal cells, immune cells, and endothelial cells through various molecular mechanisms, triggering the activation of fibroblasts and myofibroblasts (Moss et al., 2022), which eventually leads to inappropriate deposition of extracellular matrix (Kendall and Feghali-Bostwick, 2014).

Metabolic reprogramming or dysregulation of fibroblasts, epithelial cells, immune cells, and various other types of cells have been shown to be involved in the pathogenesis of pulmonary fibrosis (Ung et al., 2021). In PF, enhanced glucose metabolism affects cell differentiation, cell proliferation, autophagy, and inflammatory responses. Glucose metabolism in epithelial cells and macrophages can metabolize glucose into lactate even in the presence of oxygen, a phenomenon known as the “Warburg effect” (Liberti and Locasale, 2016; Janssen et al., 2020; Alysandratos et al., 2021). Additionally, metabolic pathways of nucleotide, lipid, or amino acid synthesis require a glycolytic intermediate (Para et al., 2019). Fatty acid oxidation is a catabolic process in which fatty acids are broken down to produce ATP (Hooff et al., 2010). It is currently considered that lipid metabolism may affect PF through several pathways, along with increased fatty acid synthesis and decreased fatty acid oxidation (Kendall and Feghali-Bostwick, 2014). Amino acids are involved in multiple metabolic pathways that are essential for cell survival, and are sources of energy, precursors to biosynthetic processes, and help maintain tissue homeostasis (Lieu et al., 2020). Most evidence suggests that alterations in amino acid metabolism during fibrosis are associated with non-essential amino acids, and increased amino acid metabolism promotes collagen synthesis and thus affects the progression of fibrosis (Gaugg et al., 2019).

This review mainly discusses recent progress in research on metabolic reprogramming of myofibroblasts, AE cells, macrophages, and endothelial cells in PF, most of which are derived from studies about IPF, with the aim of providing a comprehensive foundation for the development of targeted therapies (Tables 1, 2).

**TABLE 1:** Overview of the connection of metabolites with pro-inflammatory and pro-fibrotic pathways in Pulmonary fibrosis.

Cell type	Metabolic type	Metabolites	Associated pathway	References
Myofibroblasts	Glucose metabolism	succinic acid	Myofibroblasts differentiation by stabilizing HIF-1α	Wang et al. (2021)
		lactate	TGF-β-induced myofibroblasts differentiation	Kottmann et al. (2012)
	Lipid metabolism	LPA	Fibroblasts recruitment and vascular leak	Tager et al. (2008)
		S1P	TGF-β- and S1P-induced myofibroblasts differentiation	Huang and Natarajan, (2015)
		GPX4	TGF-β-induced myofibroblasts differentiation	Tsubouchi et al. (2019)
		PGE2	Inhibits myofibroblasts apoptosis, proliferation, and differentiation	White et al. (2005); Wettlaufer et al. (2016); Suryadevara et al. (2020)
		Lipoxins A4	Myofibroblasts dedifferentiation	Roach et al. (2015)
	Amino acid metabolism	Glycine	Collagen production	Nigdelioglu et al. (2016); Hamanaka and Mutlu (2021)
		serine	Collagen production	O'Leary et al. (2020)
		proline	Collagen production	Hamanaka et al. (2019)
		Glutamine	Myofibroblasts differentiation and collagen production	Bernard et al. (2018)
Alveolar epithelial cells	Lipid metabolism	Elovl6	Inhibits alveolar epithelial cells apoptosis and TGF-β1 expression	Sunaga et al. (2013)
	Amino acid metabolism	Glutamine	Antioxidant defense	Shaghaghi et al. (2021)
		Dimethylarginine dimethylaminohydrolase	Inhibits NOS activity	Pullamsetti et al. (2011)
Macrophages	Glucose metabolism	Lactate	TGF-β-induced myofibroblasts differentiation	Cui et al. (2021)
	Amino acid metabolism	Arginine	Collagen production	Endo et al. (2003); Niese et al. (2010); Meziani et al. (2018)

**TABLE 2:** Overview of therapeutic targets and related mechanisms in pulmonary fibrosis.

Cell type	Metabolic type	Therapeutic target	Mechanism	References
Myofibroblasts	Glucose metabolism	PFKFB3	Promoting aerobic glycolysis and lactate production	Xu et al. (2022)
		PDK	Promoting fibroblast differentiation induced by the HIF-1α/PDK1 axis	Goodwin et al. (2018)
		Subunit A of succinate dehydrogenase	Inhibiting TGF-β1-induced elevation of succinate dehydrogenase and succinate	Wang et al. (2021)
		LDH	Promoting TGF-β-induced myofibroblasts transformation	Kottmann et al. (2012)
				Judge et al. (2018)
		GLUT	Promoting α-SMA expression in lung fibroblasts	Cho et al. (2017)
				Li et al. (2022)
		Circular RNAs	CircHIPK3/miR-30a-3p/FOXK2 pathway can prevent fibroblast activation and proliferation	Chen et al. (2017)
		Caveolin-1	Inhibiting the increased expression of glycolysis and profibrotic marker proteins through p53/microRNA-34a signaling pathway	Gopu et al. (2020)
	Lipid metabolism	LPA	Promoting fibroblasts recruitment and vascular leak	Tager et al. (2008)
		S1P	Promoting TGF-β- and S1P-induced differentiation of fibroblasts to myofibroblasts	Huang and Natarajan, (2015)
		GPX4	Regulating the oxidative modification of phospholipids	Cole-Ezea et al. (2012)
		PGE2	Inhibiting a variety of myofibroblasts functions, including apoptosis, proliferation, and differentiation	Wettlaufer et al. (2016); Suryadevara et al. (2020); White et al. (2005); Penke et al. (2018); Molina-Molina et al. (2006)
				Ivanova et al. (2013)
		Lipoxins A4	Inhibiting TGF-β1-dependent of Smad2/3 nuclear translocation	Roach et al. (2015)
	Amino acid metabolism	phosphoglycerate dehydrogenase	Promoting the synthesis of collagen	Hamanaka and Mutlu, (2021)
		Phosphoserine transaminase 1	Promoting the synthesis of collagen	Jiang et al. (2020)
		PI3K	Promoting the activation of the PI3K-Akt-mTOR pathway	O'Leary et al. (2020)
		Δ1-pyrroline-5-carboxylic acid synthase	Promoting in synthesis of collagen production	Hamanaka et al. (2019)
		GLS1	Enhancing levels of glutaminolysis through SMAD3 and p38-MAPK-dependent signaling pathways	Bernard et al. (2018)
				Esguerra and Zhao, (2019)
			The activation of PPAR-γ can decrease GLS1 protein levels and enhance DEPTOR stability through the intervention of the 2-hydroxyglutarate-KDM4A axis	Miao et al. (2022)
		Nrf2	Depleting glutamate and inhibiting myofibroblast activation	An et al. (2019)
AE2 cells	Lipid metabolism	Mfsd2a	Stabilizing surfactant levels, as well as surfactant lipid composition	Wong et al. (2022)
		FASN	Promoting the expression of profibrotic genes and stabilize lung function	Jung et al. (2018)
		Elovl6	Promoting apoptosis, and reactive oxygen species, and impairing cellular uptake of long-chain FAs	Sunaga et al. (2013)
		ApoA-I	Reducing the inflammatory proteins	Kim et al. (2010)
		Mitofusin 1/2	Maintaining the stability of surfactant protein gene	Chung et al. (2019)
	Glucose metabolism	Enolase 1	Reducing glucose absorption and promoting AE2 differentiation into ciliated and secretory cells by inhibiting autophagy	Aboushousha et al. (2021)
		GLUT1	Promoting AE2 cell proliferation	Li et al. (2020)
	Amino acid metabolism	Glutamine	Supporting mitochondrial respiration and metaboliting production	Shaghaghi et al. (2021)
		Dimethylarginine dimethylaminohydrolase	Promoting NOS activity	Pullamsetti et al. (2011)
Macrophages	Glucose Metabolism	LDHA	Promoting silica exposure-induced high levels of iNOS, TNF-α, Arg-1, IL-10, and MCP1	Mao et al. (2021)
				Judge et al. (2017)
		VHL	Regulating the transcription of Spp1 *via* inhibiting HIF1α-mediated glycolytic metabolism	Zhang et al. (2018)
	Lipid metabolism	GM-CSF	Enhancing the clearance of oxidized lipids and preventing damage to anti-fibrotic molecules	Romero et al. (2015)
				Moore et al. (2000)
		Surfactant protein C	Enhancing the expression of cholesterol metabolism and transport genes	Ruwisch et al. (2020)
		FXR	Regulating intracellular lipids through targets include the B class oxidized phospholipid scavenger receptor, CD36, and the lipid chaperone, ApoE	Silverstein et al. (2010)
				Li et al. (2012)
		GPR84	Promoting phagocytosis and secreting pro-inflammatory factors	Chen et al. (2020)
				Marsango et al. (2022)
	Amino acid metabolism	Arginine	Promoting the synthesis of collagen	Niese et al. (2010)
ECs	Glucose metabolism	Ras homolog-related kinase subtype 2	Promoting intracellular pH and cell migration	Lee et al. (2020)
	Lipid metabolism	phospholipase D	Altering pulmonary vascular pathophysiology	Parinandi et al. (1999)
				Patel et al. (2011)

## Myofibroblasts

**FIGURE 1:**
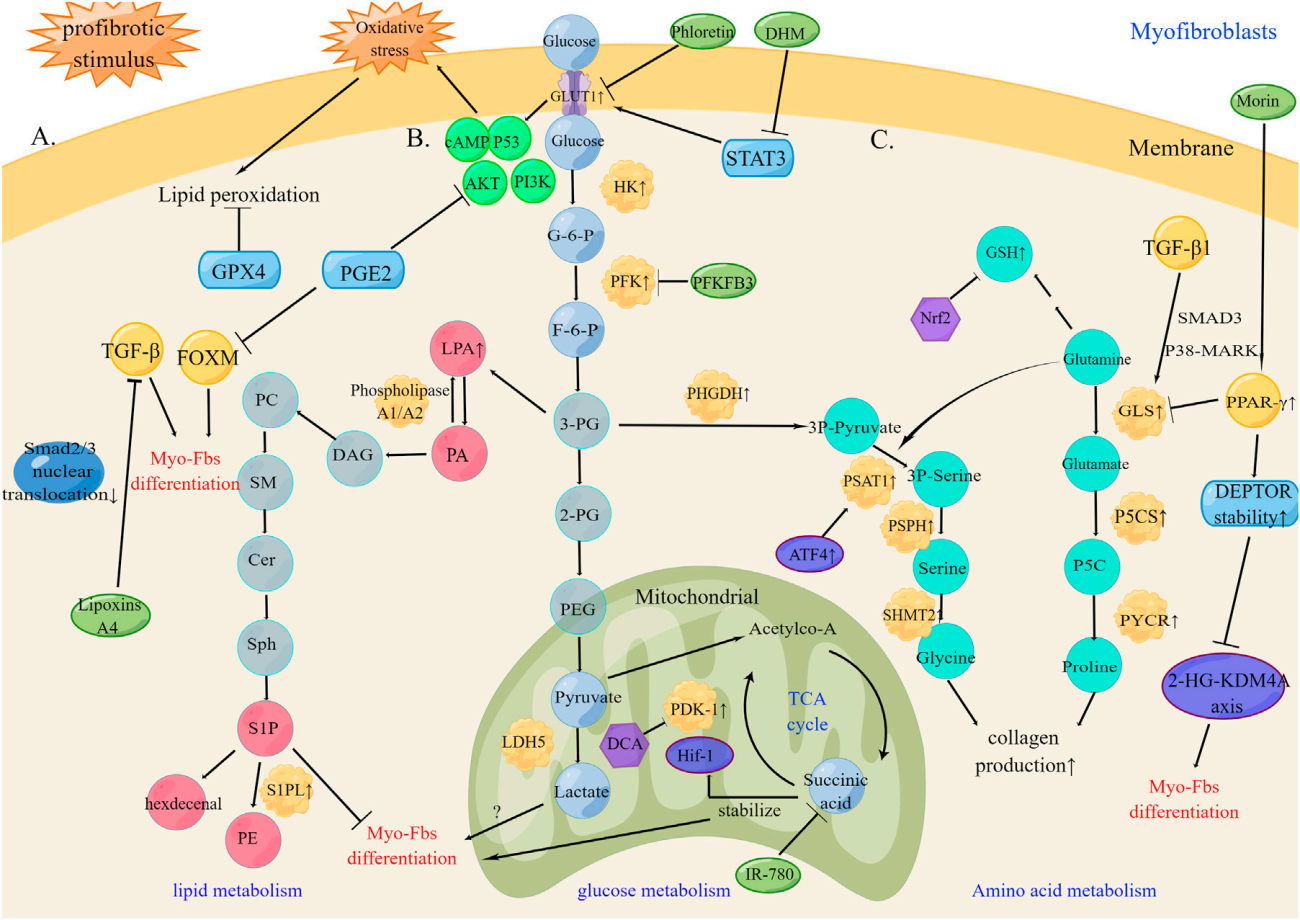
Metabolic reprogramming of myofibroblasts in pulmonary fibrosis **(A)**. Under profibrotic stimulus, lipid metabolism increases phospholipid synthesis and lipid peroxidation products. The therapeutic targets GPX4, PGE2, and TGF-β can inhibit myofibroblasts differentiation **(B)**. Under profibrotic stimulus, increased aerobic glycolysis up-regulates HK, PFK, and PDK and GLUT1 expression by changes in metabolic status and oxidative stress promote myofibroblasts differentiation into myofibroblasts by the HIF-1α/PDK1 axis. The therapeutic targets PFKB3, PDK1, and succinic acid can inhibit myofibroblasts differentiation **(C)**. Under profibrotic stimulus, amino acid metabolism promotes glutaminolysis and increases Glycine and GSH production leading to collagen production. The therapeutic targets PPAR-γ and DEPTOR stability can inhibit collagen production and myofibroblasts differentiation. PC, Polycarbonate; SM, sphingomyelin; Cer, ceramide; Sph, sphingosine; DAG, Diacylglycerol; HK, hexokinase; G-6-P, glucose-6-phosphate; PFK, phosphofructokinase; F-6-P, fructose-6-phosphate; 3-PG, 3-phosphoglycerate; 2-PG, 2- phosphoglycerate; PEG, phosphoenolpyruvate; DCA, Dichloroacetate; TCA cycle, tricarboxylic acid cycle; 3P-pyruvate, 3-phosphohydroxy-pyruvate; 3P Serine, 3-phosphohydroxy-serine; PHGDH, phosphoglycerate dehydrogenase; PSAT1, phosphorylated serine aminotransferase 1; PSPH, phosphorylated serine phosphatase; SHMT2, serine hydroxymethyltransferase 2; P5CS, Δ1-pyrroline-5-carboxylic acid synthase; PYCR, pyrroline-5-carboxylate reductase.

Key enzyme alterations in glucose, lipid, and amino acid metabolism lead to the activation and persistence of myofibroblasts in the lung tissue, which influences the progression of pulmonary fibrosis (Figure 1).

### Glucose metabolism in myofibroblasts

Increased aerobic glycolysis in lung fibroblasts promotes its differentiation into myofibroblasts (Bernard et al., 2015; Hu et al., 2020). The irreversible rate-limiting enzymes involved in glycolysis, including hexokinase, phosphofructokinase, and pyruvate dehydrogenase kinase (PDK), are all significantly up-regulated in myofibroblasts during PF. An allosteric activator of phosphofructokinase-1, 6-phosphofructo-2-kinase/fructose-2,6- biphosphatase 3 (PFKFB3) is also upregulated in Transforming growth factor-β (TGF-β)-induced myofibroblasts, and the inhibition of PFKFB3 effectively decreases levels of myofibroblasts differentiation and pulmonary fibrosis (Xie et al., 2015). PFKFB3 expression can be increased by the secretion of TNF-α in lung fibroblasts, while PFKFB3 suppression effectively blocks TNF-α-induced aerobic glycolysis and lactate production in fibroblasts (Xu et al., 2022). The anti-fibrotic effect of Anlotinib is demonstrated to inhibit PFKFB3-dependent glycolysis, which can be post-transcriptionally regulated by the RNA-binding protein PCBP3 (Chen et al., 2021).

Hypoxia is one of the prominent features in fibrotic diseases (Higgins et al., 2008). Hypoxia-inducible factor-1 (HIF-1)-mediated anaerobic glycolysis provides the metabolic reprogramming necessary for the survival of hypoxic cells (Goodwin et al., 2018). PDK1, a direct target gene of HIF-1, can be induced by TGF-β and is responsible for cellular hypoxic adaptation by enhancing glycolysis and inhibiting mitochondrial respiration (Kim et al., 2006). Dichloroacetate, a PDK1 inhibitor, can efficiently inhibit fibroblast differentiation induced by the HIF-1α/PDK1 axis (Goodwin et al., 2018). In addition, succinic acid, the metabolic product of glycolysis, has been reported to promote the differentiation of lung myofibroblasts by stabilizing HIF-1α (Wang L. et al., 2021). These studies indicate that HIF-1α is an important regulator of pulmonary fibrosis. Dichloroacetate, a potent PDK inhibitor, effectively inhibits TGF-β-mediated myofibroblast differentiation *in vitro* and induces pulmonary fibrosis *in vivo*, causing altered fibroblast metabolism (Goodwin et al., 2018). A near-infrared small molecule dye, IR-780, can target subunit A of succinate dehydrogenase in fibroblasts, and inhibit TGF-β1-induced elevation of succinate dehydrogenase and succinate, thereby preventing the formation of pulmonary fibrosis and respiratory dysfunction (Wang Z. et al., 2021).

The conversion of pyruvate to lactate is catalyzed by lactate dehydrogenases (LDHs). There is a total of five isoenzymes (Drent et al., 1996). It has been now well established from a variety of studies that lactate dehydrogenase 5 (LDH5) might play a role in TGF-β-induced myofibroblast differentiation (Kottmann et al., 2012). Gossypol, a natural non-selective LDH inhibitor, reduces TGF-β-induced myofibroblasts transformation in primary human lung fibroblasts and radiation-induced pulmonary fibrosis in mice (Judge et al., 2018). However, a recent study demonstrated that a potent small molecule inhibitor (compound 408) can inhibit LDH5 in human lung fibroblasts by reducing aerobic glycolysis and lactate production, rather than by affecting fibroblast-to-myofibroblasts differentiation (Schruf et al., 2019). Glucose transporter 1 (GLUT1) is the most conserved and widely distributed glucose transporter in mammalian cells (Thorens and Mueckler, 2010). Recent research found that GLUT1 expression is regulated by changes in metabolic status and oxidative stress, which involve signaling molecules such as cAMP, p53, phosphoinositide 3-kinase (PI3K), and AKT (Wieman et al., 2007; Zhang et al., 2013). Inhibition of GLUT1 decreases α-SMA expression in lung fibroblasts, indicating a direct link between glycolytic activation and the pro-fibrotic transformation of myofibroblasts (Cho et al., 2017). Phloretin can activate AMP-activated protein kinase *via* inhibiting GLUT1-dependent glycolysis, modulate key metabolic pathways of fibroblast activation, and significantly inhibit bleomycin-induced pulmonary fibrosis in mice (Cho et al., 2017). Dihydromyricetin can alleviate lung fibrosis in mouse models through STAT3/pSTAT3/GLUT1 signaling pathway and inhibit fibroblast or myofibroblast differentiation, proliferation, and migration (Li et al., 2022). These *in vivo* experiments provide new directions for clinical translation.

Circular RNAs, which are post-transcriptional regulators of gene expression, have been found to be dysregulated in many fibrotic diseases, such as cardiac fibrosis, renal fibrosis, liver fibrosis, and pulmonary fibrosis (Wang et al., 2019; Li J. et al., 2020; Gu et al., 2020). TGF-β1 triggers the expression of circHIPK3 in fibroblasts, enhancing FOXK2 expression by sponging miR-30a-3p. The knockdown of FOXK2 abolished increased levels of glycolytic and fibrotic markers induced by TGF-β1, while knockdown of circHIPK3 prevented fibroblast activation and proliferation (Chen et al., 2017). Therefore, strategies that target the circHIPK3/miR-30a-3p/FOXK2 pathway may provide therapeutic benefits against pulmonary fibrosis.

Deletion of Caveolin-1 in tumor-associated fibroblasts is thought to reverse the Warburg effect (Guido et al., 2012). Previous studies have shown that restoration of Caveolin-1 expression or Caveolin-1 scaffolding domain peptide-or its 7-amino acid deletion fragment Caveolin-1 scaffolding domain peptide 7-inhibited the increased expression of glycolysis and profibrotic marker proteins in fibrotic lung fibroblasts, restoring the expression of baseline p53 and its downstream transcriptional target microRNA-34a (Gopu et al., 2020). Caveolin-1 scaffolding domain peptide 7 may be a promising future therapeutic drug that can be used to inhibit uncontrolled glycolysis in fibrotic lung fibroblasts.

Senescent fibroblasts in chronic obstructive pulmonary disease (COPD) exhibit decreased glycolysis and mitochondrial respiration, leading to a great deal of ROS production and mitochondria biogenesis (Lerner et al., 2016). Heme oxygenase-1 reduces cellular and mitochondrial ROS production in COPD fibroblasts, which in turn improves mitochondrial respiration, glycolysis and ATP recovery (Even et al., 2018).

### Lipid metabolism in myofibroblasts

Lipid metabolism may affect PF *via* several pathways, including phospholipid synthesis, lipid peroxidation products, and lipid mediators. Sphingolipids emerge as potential regulators of tissue fibrosis because of their modulatory effect on cell migration, gene expression, and cell-cell interactions. In particular, lysophosphatidic acid (LPA) and sphingosine 1-phosphate (S1P) play important roles in the pathogenesis of PF (Shea and Tager, 2012; Pyne et al., 2013). LPA is a key metabolic molecule involved in the *de novo* biosynthesis of phospholipids, causes an increase in bronchoalveolar lavage (BAL) fluid levels in IPF patients compared with the controls (Tager et al., 2008; Natarajan et al., 2013). LPA is mainly generated from phosphatidic acid (PA) by phospholipase A1 or phospholipase A2 (Zhao and Natarajan, 2013). LPA1/LPA2 receptor-deficient mice are proven to be protected from bleomycin-induced pulmonary fibrosis by mediating fibroblasts recruitment and vascular leak (Tager et al., 2008). BMS-986020, a high-affinity small molecule antagonist of LPA1 and being tested in the Phase 2 Trial, improves biomarkers of fibrosis or inflammation (Palmer et al., 2018).

It is also found that sphingosine-1-phosphate (S1P), the final product of sphingolipid metabolism, is upregulated in BAL fluid of IPF patients. S1P can be irreversibly degraded by S1P lyase to hexadecenal and phosphoethanolamine (Nagahashi et al., 2014). S1P lyase over-expression attenuates TGF-β- and S1P-induced differentiation of fibroblasts to myofibroblasts, which was mediated by up-regulating autophagy in lung fibroblasts (Huang and Natarajan, 2015). Sphingosylphosphorylcholine (SPC) is a kind of sheath lipid metabolites, SPC stimulates collagen gel contraction through S1P2 receptor and Rho/Rho kinase pathway, induces the transformation of fibroblasts into myofibroblasts, and participates in airway remodeling, thus provides a new target for the intervention of COPD (Wang et al., 2014). Therefore, targeting sphingolipid metabolism provides a novel therapeutic method of ameliorating human pulmonary fibrosis.

25-hydroxycholesterol (25-HC), enzymatically produced by cholesterol 25-hydroxylase in macrophages, are increased in COPD lungs (Sugiura et al., 2012). The elevated 25-HC may contribute to fibroblasts-mediated lung tissue remodeling by promoting myofibroblasts differentiation and the excessive release of matrix metalloproteinases through the NF-kB-TGF-β-dependent pathway (Ichikawa et al., 2013).

A number of studies have demonstrated an increase in lipid peroxidation products in exhaled breath condensate and bronchoalveolar lavage fluid of patients with PF, suggesting the involvement of lipid peroxidation imbalance in the pathogenesis of PF (Montuschi et al., 1998; Kanoh et al., 2005). Glutathione peroxidases 4 (GPX4), which is commonly referred to as phospholipid hydroperoxide GPX, regulates the oxidative modification of phospholipids, including cholesteryl ester and cardiolipin (Cole-Ezea et al., 2012). Current evidence suggests that GPX4-regulated lipid peroxidation was involved in the TGF-β-induced differentiation of human lung fibroblasts (Tsubouchi et al., 2019). Reducing levels of lipid peroxidation using GPX4 may be a promising therapeutic method for PF.

Lipid mediators, also known as oxidized lipids (oxylipin), are a series of oxidative metabolites generated by the auto-oxidation of polyunsaturated fatty acids or under the action of specific enzymes (cyclooxygenase, lipoxygenases, cytochromeP450). Prostaglandin E2 (PGE2), a cyclooxygenase-2-derived eicosanoid, is an endogenously produced lipid mediator that inhibits a variety of myofibroblasts functions, including apoptosis, proliferation, and differentiation. The anti-fibrotic cascade of lung myofibroblasts regulation between PGE2 receptors 1–4 and the TGF-β/TGF-β R1/TGF-β R2 signaling pathway, while PGE2 has been reported to directionally reverse most of the genes (708 of 1277) altered by TGF-β (Wettlaufer et al., 2016; Suryadevara et al., 2020). PGE2 can also inhibit myofibroblasts differentiation through AKT dephosphorylation and elimination of PI3K signaling (White et al., 2005). Moreover, inhibition of FOXM1 expression by PGE2 can achieve myofibroblasts dedifferentiation and anti-apoptosis by interfering with the binding of FOXM1 to the promoter elements of the target genes (Penke et al., 2018). Clinical translation of PGE2 may be a promising therapy for PF patients. Losartan enhances the synthesis of PGE2 in experimental pulmonary fibrosis and has been widely used as a new treatment for pulmonary fibrosis in clinical practice (Molina-Molina et al., 2006). Inhalation of PGE2 in the form of liposomes into the lung can effectively improve pulmonary fibrosis, which is expected to become a therapeutic option for inhaled treatment of pulmonary fibrosis (Ivanova et al., 2013).

Lipoxins are also produced from membrane arachidonic acid through biochemical synthesis that involves the enzymes, 5-lipoxygenases, and 15-lipoxygenases (Bannenberg and Serhan, 2010). Lipoxins A4 inhibits various TGF-β1-dependent profibrotic responses in IPF-derived human lung myofibroblasts due to the inhibition of Smad2/3 nuclear translocation, and promotes human lung myofibroblasts dedifferentiation in the resting state (Roach et al., 2015).

### Amino acid metabolism in myofibroblasts

Numerous studies have demonstrated that collagen production by lung fibroblasts was independent of extracellular amino acids and mainly relied on the *de novo* production of amino acids (Nigdelioglu et al., 2016). Glycine is the main amino acid involved in the synthesis of collagen. During the *de novo* synthesis of glycine, the amino group provided by glutamate converts the glycolytic intermediate 3-phosphoglycerate into glycine, combined with the participation of phosphoglycerate dehydrogenase, phosphorylated serine aminotransferase 1, phosphorylated serine phosphatase and serine hydroxymethyltransferase 2 (Hamanaka and Mutlu, 2021). TGF-β induces the expression of the aforementioned enzymes, while the inhibition of *de novo* glycine synthesis caused by the phosphoglycerate dehydrogenase inhibitor, NCT503, decreases levels of bleomycin-induced lung failure in mice (Hamanaka et al., 2018).

Phosphoserine transaminase 1 is a key enzyme involved in serine synthesis (Jiang et al., 2020), and its loss is sufficient to inhibit collagen synthesis. It has been documented that TGF-β promotes the accumulation of activating transcription factor 4 (ATF4), which is required for the increased expression of serine-glycine synthesis enzymes in response to TGF-β. Furthermore, TGF-β activates the PI3K-Akt-mTOR pathway, while inhibition of PI3K prevents the activation of downstream signaling pathways and the induction of ATF4 (O'Leary et al., 2020), indicating that mTORC1 and ATF4 are important regulators of serine-glycine metabolic reprogramming in myofibroblasts.

*De novo* proline biosynthesis has also been found to be involved in pulmonary fibrosis. α-ketoglutarate, an intermediate of the citric acid cycle, is converted to glutamate by glutamate transaminase. Δ1-pyrroline-5-carboxylic acid synthase, which is encoded by the aldehyde dehydrogenase 18A1, is responsible for the conversion of glutamate to pyrrole acid, which is then converted to proline by pyrroline-5-carboxylate reductase. During the process of the *de novo* conversion of proline from glutamate, its knockout is sufficient to inhibit collagen production in lung fibroblasts (Hamanaka et al., 2019).

Glutaminolysis has been recently shown to be required for myofibroblast differentiation and collagen production (Bernard et al., 2018). Glutamine is first converted to glutamate by glutaminase (GLS) and then converted to α-ketoglutarate by glutamate dehydrogenase or aminotransferases. This process complements the Krebs cycle, which supports cellular energy production and biosynthetic reactions (Altman et al., 2016). TGF-β1-induced glutaminolysis underlies the persistent activation of HIF-1 by increasing succinate and fumarate levels, thereby supporting the glycolytic switch. TGF-β1 induces GLS1 expression *via* SMAD3 and p38-MAPK-dependent signaling pathways, which are accompanied by enhanced levels of glutaminolysis in myofibroblasts. The GLS1 inhibitor, CB-839, exerts a protective effect in mice exposed to bleomycin or adenovirus expressing active TGF-β1 (Bernard et al., 2018; Esguerra and Zhao, 2019). Additionally, PPAR-γ is a regulator of GLS1-mediated glutaminolysis (Genovese et al., 2005). Morin (3, 5, 7, 2, 4-pentahydroxyflavonoid) has recently been shown to inhibit the transformation of fibroblasts to myofibroblasts by activating PPAR-γ, decreasing GLS1 protein levels, and inhibiting glutaminolysis, thereby enhancing DEPTOR stability through the intervention of the 2-hydroxyglutarate-KDM4A axis (Miao et al., 2022). The glutamine metabolite, 2-hydroxyglutarate (Chowdhury et al., 2011; Carbonneau et al., 2016), is a specific inhibitor of Jumonji domain-containing histone demethylases activity. DEPTOR is extremely unstable, whereas catalytically active KDM4A, a Jumonji domain-containing histone demethylases, negatively regulates mTORC1 activation. Glutaminolysis-related mTOR activation in organ fibrosis not only regulates collagen expression but also controls autophagy and mitochondrial levels in myofibroblasts (Ge et al., 2018). Notably, the mTOR inhibitor, rapamycin, attenuates inflammatory cell infiltration and collagen deposition, thereby antagonizing PF (Gui et al., 2015). In addition to rapamycin, some studies have found that Morin, a natural flavonoid, affects mTOR1 to achieve anti-fibrosis.

Cytosolic glutamate is critical for maintaining redox balance and protecting cells from oxidative stress by producing glutathione (GSH) (Chen and Cui, 2015). Nuclear factor-erythroid 2-related factor 2 (Nrf2) binds to the antioxidant response elements and stimulates the transcription of antioxidant proteins, as well as proteins involved in GSH biosynthesis and regeneration. Increased GSH production by Nrf2 activation may inhibit myofibroblast activation by limiting glutamate availability for cell growth (Motohashi and Yamamoto, 2004). Tanshinone IIA, a Chinese herbal medicine derived from Salvia miltiorrhiza, inhibits fibrotic responses and TGF-β1-dependent epithelial-to-mesenchymal transition in pulmonary fibrosis. Tanshinone IIA activation of Nrf2 depletes glutamate involved in GSH synthesis, thereby inhibiting myofibroblast activation (An et al., 2019). An individual’s ability to regulate antioxidant defense mechanisms, such as GSH levels in response to cigarette smoke, has also been linked to the development of COPD in smokers (Snider, 1989). Smoke inhibits fibroblasts repair, reflected in collagen gel contraction and fibronectin production, possibly regulated by intracellular GSH levels (Kim et al., 2002).

## Alveolar epithelial cells

**FIGURE 2:**
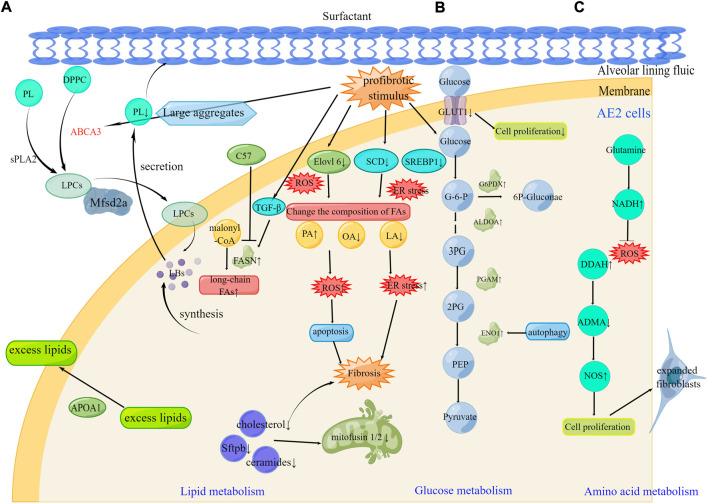
Metabolic reprogramming of AE2 cells in pulmonary fibrosis **(A)**. Alteration of FAs composition leading to ER stress and ROS, abnormal lipid uptake on the lung surface, and reduced lipid production caused by mitochondrial destruction all contribute to fibrosis progression. The therapeutic targets Mfsd2a, FASN, and ApoA1 can be effective in the development of pulmonary fibrosis **(B)**. Under profibrotic stimulus, increased PGAM, ENO1, ALDOA, and G6PDX affect glucose metabolism. The therapeutic targets GLUT1 and ENO1 can be effective in the development of pulmonary fibrosis **(C)**. Under profibrotic stimulus, increased DDAH activity inhibited the NOS inhibitor ADMA contributing to the pool of expanded fibroblasts. The therapeutic targets glutamine and DDAH can be effective in the development of pulmonary fibrosis. DPPC, Dipalmitoylphosphatidylcholine; sPLA2, secreted phospholipase 2; LPCs, lysophosphatidylcholines; LBs, Lamellar bodies; SCD, stearyl CoA desaturase; SREBP1, sterol regulatory element-binding protein 1; ER stress, endoplasmic reticulum stress; FA, Fatty acid; PA, Palmitic acid; OA, oleic acid; LA, linoleic acid; G6PDX, glucose-6-phosphate dehydrogenase; ALDOA, aldolase A fructose bisphosphate; PGAM, phosphoglycerate mutase; ENO1, enolase 1; DDAH, dimethylaminohydrolase; ADMA, asymmetric dimethylarginine.

Alveolar epithelial (AE) cells include alveolar epithelial type I (AE1) and alveolar epithelial type II (AE2) cell types that integrate into the epithelium at extensive intercellular junctions (Ruaro et al., 2021). AE2 cells are the major driver of PF pathogenesis (Winters et al., 2019). Dysregulation of lipid, glucose, and amino acid metabolism in AE2 cells have been demonstrated during the development of PF (Figure 2).

### Lipid metabolism in alveolar epithelial type II cells

*De novo* lipid synthesis, an important feature of AE2 cells, is required to generate lipid-rich surfactants, by which respiratory epithelial cells can be soaked in by reducing surface tension and resisting environmental challenges (Ingenito et al., 2000; Schmidt et al., 2001). The reprogramming of lipid metabolism in AE2 cells is one of the main mechanisms that lead to PF.

Dipalmitoylphosphatidylcholine, the main pulmonary surfactant phospholipid (PL) secreted by AE2 (Pérez-Gil, 2008), is synthesized by fatty acid acyl chain remodeling *via* the cyclic activity of Land on unsaturated fatty acids (FAs). Dipalmitoylphosphatidylcholine is then transported to lysosomes called lamellar bodies *via* the surfactant transporter ABCA3 like organelles. Lamellar bodies are secreted into large aggregated membrane bilayers and are rapidly absorbed into the PL monolayers to form lung surfactant films. Through the ventilation process, the surfactant film becomes disordered, and the surfactant PL has also been shown to be hydrolyzed by secreted phospholipase in the alveolar space to generate lysophosphatidylcholines (Bernhard et al., 2004; Ban et al., 2007). Previous studies have found that mutations in ABCA3, such as the most common missense mutation E292V, lead to the development of IPF. Mutations in ABCA3 result in chronic surfactant insufficiency and decrease PL in bronchoalveolar lavage (Beers et al., 2017). Inhibition of Mfsd2a in AE2 cells results in hypertrophy, a lack of steady-state surfactant levels, and alteration in surfactant lipid composition (Wong et al., 2022). Since Mfsd2a plays an important role in maintaining surfactant homeostasis, it remains to be determined whether it can play a role in pulmonary fibrosis.

Fatty acid synthase (FASN) is an essential anabolic enzyme for the *de novo* synthesis of FAs (Menendez and Lupu, 2007). FASN catalyzes the reduction of malonyl-CoA to synthesize long-chain fatty acids (Chung et al., 2019). TGF-β has been reported to increase FASN expression in mouse and human fibroblasts in a smad2/3-dependent, mTORC1-dependent manner. Most importantly, cyanin-derived FASN inhibitor C75, reduces the expression of profibrotic genes and stabilizes lung function (Jung et al., 2018). Recent studies performed mRNA sequencing of lung tissue obtained from surgical lung biopsies of patients with early IPF and IPF during transplantation, and have verified that FASN is downregulated in AE cells, ciliated cells, and alveolar macrophages in IPF lung tissue (Qian et al., 2021). Moreover, the loss of AE2 cell-specific FASN results in a reduction of lipid synthesis and aggravates pulmonary fibrosis induced by bleomycin (BLM). The role of FASN during lipid metabolism in pulmonary fibrosis needs to be explored further.

Elongation and desaturation of FAs from the core of lipid biosynthesis determines their function and metabolic fate. Enzymes that are involved in FA elongation and saturation, such as stearyl CoA desaturase, and related molecules, such as sterol regulatory element-binding protein 1, have been reported to be down-regulated during the development of PF, with endoplasmic reticulum stress playing an important role in the downregulation. The elongation of long-chain FAs family member 6 (Elovl6), an important rate-limiting enzyme of lipid biosynthesis, catalyzes the extension reaction of palmitate (C16:0) to stearate (C18:0). The loss of Elovl6 induces apoptosis and the production of reactive oxygen species, and most importantly, impairs cellular uptake of long-chain FAs (Sunaga et al., 2013). Elovl6-mediated FA metabolism in AE2 cells is perturbed in models of pulmonary fibrosis, which may have important implications for lung pathology and therapy.

Lipid synthesis is regulated not only by lipid synthases but also by other molecular mechanisms. Apolipoprotein A1, the most abundant protein in high density lipoprotein, has recently been found to be potentially increased in the lung tissue by alveolar epithelial cells and macrophages, to remove excess lipids from cells to prevent lipid overload. ApoA1 exerts a protective effect against lung injury and fibrosis (Kim et al., 2010; Eh et al., 2013; Gordon et al., 2016).

Emerging evidence suggests that mitochondrial disturbances and reduced levels of energy production lead to the disturbance of various metabolic processes (Shaghaghi et al., 2021). The mitochondrial damage-induced loss of mitofusin 1 and mitofusin 2, GTPase proteins that coordinates the fusion of the mitochondrial outer membrane, abolishes the lipogenic metabolic responses of the AE2 cells, which finally leads to pulmonary fibrosis (Chung et al., 2019). Dysregulated lipogenic metabolic responses include the reduced production of liposomes, such as cholesterol and ceramides, and the significant down-regulation of surfactant protein genes (Sftpb, Sftpc). Overall, AE2 lipid metabolism deficiency drives pulmonary fibrosis, providing an innovative mechanism and potential pharmacological target.

### Glucose metabolism in alveolar epithelial type II

Aerobic glycolysis and pentose phosphate pathways are essential for AE2 cell activation, proliferation, and regeneration (Li et al., 2019). During the early stage of PF, the lactose levels are elevated in AE2 cells due to the “Warburg effect”, which then induces an increase in TGF-β expression that promotes fibrosis. However, during the middle and late stages, the levels of most glycolytic metabolites are decreased in AE2 cells (Roque and Romero, 2021). Recent studies have shown that the expression of key glycolytic enzymes, such as phosphoglycerate mutase, enolase 1, and aldolase A fructose bisphosphate are elevated in PF. The rate-limited enzyme glucose-6-phosphate dehydrogenase of the pentose phosphate pathway is also upregulated. Autophagy is responsible for alterations of enolase 1 levels during AE2 cell proliferation and the regulation of lung homeostasis in PF (Li X. et al., 2020). Inhibition of autophagy reduces glucose uptake, and glucose deprivation or blockade of glycolysis reduces the proliferative capacity of AE2 cells and promotes their differentiation into ciliated and secretory cells (Aboushousha et al., 2021). In addition to the above-mentioned metabolic enzymes that lead to enhanced glucose metabolism, GLUT1 also plays an important role. During bleomycin-induced injury, conditional deletion of GLUT1 reduces AE2 cell proliferation and impairs AE2 cell recovery *in vivo*. Under this injury condition, the glycolysis and pentose phosphate pathways are enhanced in AE2 cells (Li J. et al., 2020). Thus, the role of aerobic glycolysis and pentose phosphate pathways in AE2 cells in pulmonary fibrosis provides a basis for a better understanding of pulmonary homeostasis and injury repair.

### Amino acid metabolism in alveolar epithelial type II

The demand for amino acids increases during various pathological conditions, including pulmonary fibrosis (Gaugg et al., 2019). Glutamine is involved in antioxidant defense through multiple mechanisms, including the production of NADPH to regulate the synthesis of various ROS detoxification enzymes and the core protein of cellular antioxidant defense glutathione. It has been demonstrated that a profound metabolic shift occurs during glucose metabolism, requiring cells to rely on other metabolic fuels, such as glutamine, to support mitochondrial respiration and metabolite production, thereby inhibiting the progression of pulmonary fibrosis (Shaghaghi et al., 2021). Mechanically, diminished mitochondrial respiratory function in pulmonary fibrosis can be restored by adding glutamine to the culture medium of AE2 cells. Except for directly increasing the amino acid content, enzymes that regulate amino acid metabolism are equally important for protection against pulmonary fibrosis. Dimethylarginine dimethylaminohydrolase metabolizes asymmetric dimethylarginine and monomethylarginine to l-citrulline and dimethylamine, both of which are endogenous inhibitors of NOS (Pullamsetti et al., 2011). Dimethylarginine dimethylaminohydrolase inhibitors increase intracellular asymmetric dimethylarginine concentrations to levels that are sufficiently to inhibit NOS activity, resulting in the inhibition of pulmonary fibrosis progression. Together, these studies underlie the important role of amino acid metabolism and may offer possible therapeutic avenues for the attenuation of pulmonary fibrosis.

## Macrophages

Pulmonary macrophage populations are mainly composed of alveolar macrophages (AMs) and interstitial macrophages (IMs), which reside in the alveoli and parenchyma respectively (Guilliams et al., 2013). In addition, pulmonary macrophages can also change their phenotype through polarization into either classically activated macrophages (M1) or alternatively activated macrophages (M2), which play important roles in homeostasis and tissue remodeling in PF (Locati et al., 2013).

### Glucose metabolism in macrophages

M1 macrophages mainly rely on glycolysis for energy and pro-inflammatory effects, while M2 macrophages preferentially utilize oxidative phosphorylation to promote repair of pulmonary tissue damage (Viola et al., 2019). Enhanced glycolysis in alveolar macrophages obtained from fibrotic lungs is caused by the increased expression of important glycolytic mediators or enzymes induced by the M1 activator, IFN-γ, and LPS, such as GLUT1, hexokinase 2, phosphofructokinase liver type and LDHB (Xie et al., 2017). However, these molecules are not induced by the M2 inducers, IL-4, IL-10, or TGF-1. Moreover, inhibition of glycolysis mitigates the increase in the expression of M2 phenotype markers in alveolar macrophages obtained from fibrotic lungs (Xie et al., 2017).

Except during the inflammatory response, macrophages also rely on glycolysis to produce high levels of lactate (Soto-Heredero et al., 2020), which sustain a low pH in the extracellular space and activate TGF-β1-induced fibrotic signaling (Kottmann et al., 2012). The silencing of LDHA, the enzyme that catalyzes lactate production, can suppress silica exposure-induced high levels of iNOS, TNF-α, Arg-1, IL-10, and MCP1 in NR8383 alveolar macrophages (Mao et al., 2021). Mechanically, lactate can increase histone lactylation at the profibrotic gene promoters of macrophages (Cui et al., 2021), providing a novel therapeutic target for PF. The regulation of lactate level can be accomplished by directly blocking lactate production in lung macrophages, or by depleting extracellular lactate, thereby reducing lactate entry into lung macrophages and other cell types. The LDHA inhibitor gossypol inhibits ionizing radiation- and bleomycin-induced pulmonary fibrosis (Judge et al., 2017). Oxalate, a competitive inhibitor of LDHA, has likewise been shown to maintain macrophage homeostasis by ameliorating the increase in glycolysis and endoplasmic reticulum stress induced by the exposure of macrophages to silica (Mao et al., 2022).

**FIGURE 3:**
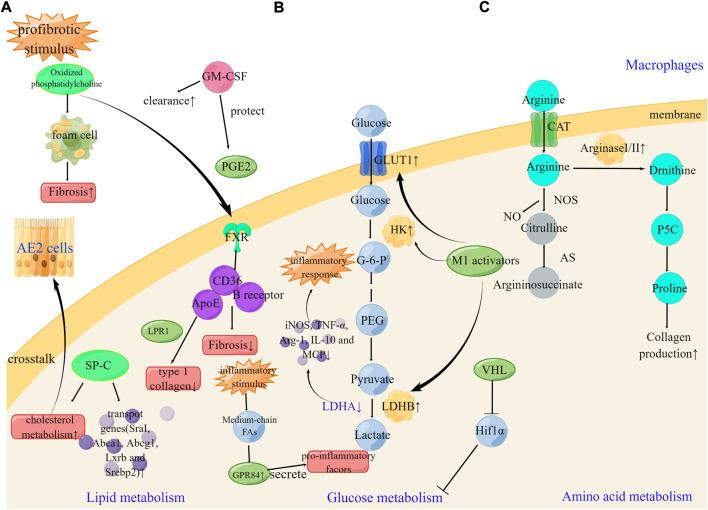
Metabolic reprogramming of macrophages cells in pulmonary fibrosis **(A)**. GM-CSF, SP-C, and FXR can enhance the clearance of oxidized lipids and regulate lipid metabolism. The therapeutic targets FXR, ApoE and GPR84 can be effective in the development of pulmonary fibrosis **(B)**. Under profibrotic stimulus, M1 activators increase GLUT1, hexokinase, and LDHB leading to enhance glucose metabolism. Silencing of LDHA can suppress high levels of iNOS, TNF-α, Arg-1, IL-10, and MCP1. The therapeutic targets LDHA and VHL can be effective in the development of pulmonary fibrosis **(C)**. Arginase metabolism is partially dependent on the transport of arginine through CAT2 in peritoneal macrophages. The therapeutic targets Arginase can be effective in the development of pulmonary fibrosis. SP-C, surfactant protein C; LPR1 lipoprotein receptor–related protein 1; B receptor; B class oxidized phospholipid scavenger receptor.

Under normoxia, HIF1α is kept at low levels due to the recognition and ubiquitination of E3 ligase von Hippel-Lindau (VHL) and the ensuing proteasomal degradation (Semenza, 2011). Loss of VHL results in an upshift in glycolysis, impairs mitochondrial respiration and alters gene expression characteristics in the AMs. VHL regulates the transcription of Spp1, a hypoxia-responsive gene in tumor cells, *via* inhibiting HIF1α-mediated glycolytic metabolism in AMs (Zhang et al., 2018), indicating that VHL is a key regulator of alveolar macrophages in PF (Figure 3).

Metabolic dysfunction is a key factor in the toxicity of cigarette smoke on alveolar macrophages. Cigarette smoke inhibits mitochondrial respiration in alveolar macrophages while inducing glycolysis and reactive oxygen species. These defects were alleviated by the free radical scavenger N-acetylcysteine, which also restored phagocytosis to baseline levels (Aridgides et al., 2019). CPUY192018, a nuclear factor erythrocyte-2-related factor 2 (Nrf2) activator, can inhibit glycolysis, enhance antioxidant stress, and produce satisfactory therapeutic effects on macrophages of COPD patients and cigarette smoke extract-induced COPD mice, which is expected to be a potential treatment for COPD (Wang L. et al., 2021). These studies provide a further fundamental mechanistic basis for the use of antioxidants in COPD.

### Lipid metabolism in macrophages

Profibrotic injury-induced early and persistent metabolic changes in AE2 cells, which then lead to the inhibition of lipid synthesis and the release of stored lipids into the distal airspaces of the lung. Subsequently, the extracellular accumulation of oxidized lipids promoted the development of macrophage foam cells, which results in a fibrotic cascade (Ingenito et al., 2000; Schmidt et al., 2001; Forbes et al., 2007). Granulocyte-macrophage colony-stimulating factor (GM-CSF) is a 23-kDa glycoprotein member of the hematopoietic cytokine family that regulates the proliferation and differentiation of cells of the granulocyte-macrophage lineage (Baldwin, 1992). Recently, GM-CSF has been shown to improve pulmonary fibrosis *via* regulating lipid metabolism. Oxidized phosphatidylcholine is sufficient to induce foam cell formation and can promote fibrosis in mice, while GM-CSF treatment can enhance lipid clearance in macrophages (Romero et al., 2015). PGE2 exerts a protective effect on fibroblasts as previously mentioned, and GM-CSF deficiency leads to an enhanced level of fibrosis in bleomycin-induced pulmonary fibrosis, with a mechanism of action involved in this effect identified as the impaired production of potent anti-fibrotic PGE2 in macrophages (Moore et al., 2000). GM-CSF not only enhanced the clearance of oxidized lipids in macrophages but also prevented damage to anti-fibrotic molecules. PGD2, another COX metabolite, is produced in CD68-positive monocytes/macrophages, and may inhibit immune cell infiltration in an autocrine or paracrine manner by inhibiting inflammation in bleomycin-induced pulmonary fibrosis (Kida et al., 2016). Pharmacological approaches that enhance lipid efflux or prevent foam cell formation may be promising for preventing or treating pulmonary fibrosis.

A multitude of evidence has indicated the key role of surfactant protein C deficiency for the pathogenesis of IPF (Ruwisch et al., 2020; Sehlmeyer et al., 2020). Increased cholesterol levels in mouse lung tissue after bleomycin treatment have been attributed to paracrine lipid crosstalk between alveolar epithelial cells and alveolar macrophages. Preliminary observations suggest that this crosstalk may be impaired by the absence of surfactant protein C. The addition of surfactant protein C to cholesterol-containing lipid vesicles enhances the expression of cholesterol metabolism and transport genes (such as Pparg, SraI, Abca1, Abcg1, Lxrb and Srebp2) in alveolar macrophage cell lines (Ruwisch et al., 2020), which is a potential novel lipid-protein axis involved in lung remodeling in PF.

Apolipoprotein E (ApoE) is produced almost exclusively by monocyte-derived Alveolar macrophages. The expression of ApoE is significantly increased and enriched in dense fibrotic regions of IPF lungs. ApoE depends on lipoprotein receptor–related protein 1 to promote macrophage phagocytosis of type 1 collagen (Cui et al., 2020).

Several transcription factors and fatty acid receptors regulate lipid homeostasis in macrophages and have been implicated in the progression of pulmonary fibrosis. Nitrogen mustard, a known lung-targeting vesicular agent, can cause acute injury and fibrosis, and activated macrophages have been demonstrated to contribute to the pathological response to nitrogen mustard. Nitrogen mustard-induced changes sustained oxidative stress, and are mediated by Nrf2 and iNOS signaling pathways, which are associated with the accumulation of oxidized phospholipids in lung macrophages and epithelial cells (Venosa et al., 2019). The farnesoid-X receptor (FXR) has been hypothesized to regulate intracellular lipids. The number of FXR-positive macrophages in the lung is increased after nitrogen mustard administration and may represent a compensatory attempt to limit the fibrotic process. The FXR agonist, obeticholic acid, inhibited the development of bleomycin-induced fibrosis, potentially due to the attenuated production of key inflammatory mediators. At present, putative FXR targets include the B class oxidized phospholipid scavenger receptor, CD36, and the lipid chaperone, ApoE (Silverstein et al., 2010; Li et al., 2012).

G-protein-coupled receptor 84 (GPR84), the fifth most recognized fatty acid receptor, is involved in initiating Gi signaling, resulting in reduced cAMP production through the inhibition of adenylate cyclase. Medium-chain fatty acids with a chain lengths of C9-C14 are GPR84 agonists. After acute inflammatory stimulation, Medium-chain fatty acids activated GPR84 to promote phagocytosis and secrete pro-inflammatory factors (Chen et al., 2020; Marsango et al., 2022). Current research has demonstrated that GPR84 antagonists have great potential for the treatment of pulmonary fibrosis. However, further studies are needed to determine whether clinical translation can be achieved (Figure 3).

### Amino acid metabolism in macrophages

Arginine is a precursor for proline and polyamine synthesis, and since proline is an important component of collagen, the supply of proline may be a key factor during the process of pulmonary fibrosis. Arginine metabolizing enzymes are involved in the production of proline or polyamines (Durante et al., 2001; Ignarro et al., 2001). Arginase exists as two subtypes, the hepatic (arginase I) subtype and the extrahepatic (arginase II) subtype. Previous studies have emphasized that both arginase I and II are induced during bleomycin-induced pulmonary fibrosis. Arginase I is induced in macrophages, whereas arginase II is induced in a variety of cells, including macrophages and myofibroblasts. Arginine is transported into the intracellular space by cationic amino acid transporters (CATs) and is a precursor for the synthesis of urea, polyamines, phosphocreatine, nitric oxide, and proteins (Endo et al., 2003). More importantly, arginase activity is partially dependent on the transport of arginine through CAT2 in peritoneal macrophages. Therefore, arginase may play a role in CAT2-mediated pulmonary fibrosis (Niese et al., 2010), emphasizing the role of amino acid metabolism reprogramming in pulmonary fibrosis. At the steady state, IMs express 10-fold more arginase I than AMs, while Arg-140-fold upregulation has been detected in IMs isolated from radiation-induced lung fibrosis. The expression of colony-stimulating factor receptor-1 was mainly expressed on IMs, and the use of anti-CSF1R neutralizing antibody results in the specific depletion of pro-fibrotic IMs, without affecting the number of AMs, and can also reverse radiation-induced lung fibrosis (Meziani et al., 2018) (Figure 3).

## Endothelial cells

Early observations of pulmonary fibrosis show that vascular abnormalities, including pulmonary system anastomosis and neovascularization around the fibrotic area (Hamada et al., 2007; Ley et al., 2011), along with the metabolic disturbance of ECs play an important role (Lee et al., 2020).

### Glucose metabolism in endothelial cells

Pulmonary microvascular endothelial cells (PMVECs) are highly glycolytic due to their rapid growth rate (Parra-Bonilla et al., 2010). This active form of glycolysis induces extracellular acidosis, which inhibits PMVECs migration, while glycolysis acts as a negative feedback mechanism. KD025 (SLx-2119) is a relatively new inhibitor of Ras homolog-related kinase subtype 2. Studies have shown that KD025 shifts the PMVEC metabolic pathway from glycolysis to oxidative phosphorylation in a Ras homolog-related kinase subtype 2-independent manner, reduces intracellular pH and cell migration, and enhances the integrity of the lung endothelial barrier *in vivo* (Lee et al., 2020). Currently, KD025 is being tested in multiple Phase II clinical trials for use in the treatment of fibrotic lung disease, including idiopathic pulmonary fibrosis.

The role of HIF-1 in the reprogramming of glucose metabolism in pulmonary fibrosis has been previously mentioned, and identification of the switch from HIF-1 to HIF-2 in endothelial cells (EC) can ensure the continued activity of the hypoxia-adaptive pathway, thereby prolonging the hypoxia survival time. The conversion of HIF-1 to HIF-2 was disturbed by the stabilization of HIF1α and endothelial PAS domain protein 1 mRNAs in EC, which then induced the reprogramming of glucose metabolism. Exploiting the regulation of endothelial cell adaptation to hypoxia may be a potential therapeutic intervention against pulmonary fibrosis (Bartoszewski et al., 2019).

### Lipid metabolism in endothelial cells

IL-6 is a pro-inflammatory cytokine produced by endothelial cells and is actively involved in local vascular inflammatory responses (Podor et al., 1989). Previous studies have investigated the intracellular fatty acid composition modulated the production of IL-6, which is elevated in bleomycin-induced lung endothelial cells in a time-dependent and dose-dependent manner. In other words, endothelial IL-6 production may be upregulated by 18:2 n-6 and/or its derived metabolites, although this process is not directly dependent on prostacyclin or other cyclooxygenase metabolites. It has been currently established that a diet lower than 18:2 n-6 can exert a protective effect against bleomycin-induced pulmonary fibrosis (Karmiol et al., 1993). However, the fatty acid effects exerted by lipoxygenase and/or cyclooxygenase metabolites require further studies to confirm whether they can be used as a novel method of treatment for PF.

Oxidative stress-induced activation of phospholipase D and related lipid signaling enzymes are key factors involved in ECs’ dysfunction, leading to altered pulmonary vascular pathophysiology during oxidative exposure in pulmonary hypertension and pulmonary fibrosis (Parinandi et al., 1999). In addition, bleomycin-induced cytotoxicity of bovine lung microvascular ECs may be due to the signaling cascade involving PA and other PA-derived signaling mediators, such as LPA and diacylglycerol (Patel et al., 2011). Therefore, the phospholipase D pathway in vascular endothelial cells may be a potential therapeutic target for PF.

## Perspectives

Previous studies have found that men have a 40% higher risk of transplant or death than women in pulmonary fibrosis (PF) (Zaman et al., 2020). Higher smoking rates in men may account for some of the differences in gender (Ley and Collard, 2013), while mutations in telomerase and surfactant proteins associated with PF fared better in women than in men (Pandit et al., 2020). Whether there is some metabolic difference related to gender in the development of pulmonary fibrosis needs further exploration. With the advent of novel and cost-effective metabolite profiling techniques, metabolic reprogramming has been found to occur during the pathogenic process of pulmonary fibrosis. In this review, we showed that glucose metabolism is enhanced in fibroblasts, macrophages, and vascular endothelial cells, but inhibited in epithelial cells, which results in the targeting of glycolysis during pulmonary fibrosis. The enzymes and related molecular pathways were found to be therapeutically advantageous. Lipid metabolism is mainly characterized by increased lipid synthesis and fatty acid oxidation in fibroblasts, macrophages, and vascular endothelial cells. In epithelial cells, lipid metabolism is inhibited at an early stage, resulting in the accumulation of lipid oxidation. Enzymes, key molecular pathways of fatty acid oxidation, and lipid mediators form the core of currently available treatment methods. Amino acids, which are the main components of collagen, show increased levels of synthesis or decreased levels of decomposition in fibroblasts, macrophages, and epithelial cells. Therefore, the targeting of important enzymes in amino acid metabolism may be a main therapeutic strategy. Future research is required to promote the development and translation of drugs that target the metabolic disturbance in PF.
